# Identification of molecular characteristics of FUT8 and alteration of core fucosylation in kidney renal clear cell cancer

**DOI:** 10.18632/aging.205482

**Published:** 2024-01-25

**Authors:** Zhu Xin, Xinyu Wen, Mengying Zhou, Hongli Lin, Jia Liu

**Affiliations:** 1 Department of Nephrology, The First Affiliated Hospital of Dalian Medical University, Key Laboratory of Kidney Disease of Liaoning Province, The Center for the Transformation Medicine of Kidney Disease of Liaoning Province, Dalian, China; 2 Liaoning Laboratory of Cancer Genomics and Epigenomics, College of Basic Medical Sciences, Dalian Medical University, Dalian, China

**Keywords:** kidney renal clear cell cancer, core fucosylation, FUT8, tumor microenvironment, prognosis

## Abstract

Background: Kidney renal clear cell cancer (KIRC) is a type of urological cancer that occurs worldwide. Core fucosylation (CF), as the most common post-translational modification, is involved in the tumorigenesis.

Methods: The alterations of CF-related genes were summarized in pan-cancer. The “ConsensusClusterPlus” package was utilized to identify two CF-related KIRC subtypes. The “ssgsea” function was chosen to estimate the CF score, signaling pathways and cell deaths. Multiple algorithms were applied to assess immune responses. The “oncoPredict” was utilized to estimate the drug sensitivity. The IHC and subgroup analysis was performed to reveal the molecular features of FUT8. Single-cell RNA sequencing (scRNA-seq) data were scrutinized to evaluate the CF state.

Results: In pan-cancer, there was a noticeable alteration in the expression of CF-related genes. In KIRC, two CF-related subtypes (i.e., C1, C2) were obtained. In comparison to C2, C1 exhibited a higher CF score and correlated with poorer overall survival. Additionally, the TME of C2 demonstrated increased activity in neutrophils, macrophages, myeloid dendritic cells, and B cells, alongside a higher presence of silent mast cells, NK cells, and endothelial cells. Compared to normal samples, higher expression of FUT8 is observed in KIRC. The mutation of SETD2 was more frequent in low-FUT8 samples while the mutation of DNAH9 was more frequent in high-FUT8 samples. scRNA-seq analyses revealed that the CF score was predominantly higher in endothelial cells and fibroblast cells.

Conclusions: Two CF-related subtypes with distinct prognosis and TME were identified in KIRC. FUT8 exhibited elevated expression in KIRC samples.

## INTRODUCTION

Over the past few years, there has been a yearly rise in the frequency of kidney cancer cases, constituting 2.2% of newly diagnosed cancer incidents and contributing to approximately 1.8% of fatalities attributed to cancer [1, 2]. Kidney renal clear cell carcinoma (KIRC), a prevalent form of urinary system malignancy, makes up nearly 80% of all cases of renal cell carcinoma (RCC) [3–6]. Advanced KIRC is resistant to both chemotherapy and radiotherapy and it always combines with extremely poor prognosis [7]. Currently, the primary approach in treating individuals with KIRC involves surgical intervention. However, almost 40% of these patients experience metastases following initial removal [8, 9]. In addition, 25% of the KIRC patients are diagnosed with metastasis at the first visit due to no specific features at the early stage [10, 11]. As for metastatic renal cell carcinoma, only few first-line drugs such as Sunitinib can be used, but drug resistance often occurs after 6-15 months of systematic treatment [12]. Identifying key driver genes related to progression can help improve the prognosis. Besides, an in-depth exploration of the molecular mechanisms of KIRC is significant for the creation of novel therapeutic agents.

Fucosylation denotes the act of attaching a fucose molecule to glycolipids, O-glycans, and N-glycans. This process can be categorized into core fucosylation and terminal fucosylation. Being the prevalent post-translational alteration, core fucosylation (CF) participates in a range of biological mechanisms, including cell proliferation and differentiation. The creation of fucosylated glycans is associated with a set of fucosyltransferases (FUTs) that have been recognized within the human genome. Fucosyltransferase 8 (FUT8) possesses the ability to facilitate the incorporation of α-1,6-fucose onto the innermost GlcNAc unit of N-glycans. It has been established as the principal orchestrator of core fucosylation [13]. The disordered expression of fucosyltransferases (FUTs) have a close relationship with oncogenesis [14, 15]. Also, tumor immune response is revealed to be linked to aberrant core fucosylation [16]. Large amounts of immune system molecules are core fucosylated glycoproteins which participate in signal transduction, antigen clearance, and lymphocyte activation. The CF of IgG-BCR is essential in the process of antigen recognition and antibody production [17]. T cell receptors (TCRs) are core-fucosylated glycoproteins and the activation of T cell is related to the CF of the TCR [18]. In addition, most CF-related genes can positively regulate the expression of PD-1 and anti-tumor immune responses can be improved after obstructing the core fucosylation of PD-1 [19].

Absolutely, targeting the abnormal fucosylaion will be a novel strategy in cancer treatment. Till now, the abnormal CF has been found in many cancers including lung cancer [20], thyroid carcinoma [21], and hepatocellular carcinoma [22]. Whether CF functions in the tumorigenesis and progression in KIRC is poorly investigated. In this research, we systematically summarized the alteration of CF-related genes in a pan-cancer level and focused on the potential mechanisms of CF in KIRC. Using the expression levels of central core fucosylation (CF) genes, KIRC samples were categorized into two subtypes, each displaying unique traits such as survival probabilities, tumor microenvironment (TME) characteristics, and responsiveness to drugs. FUT8, as the gene of great importance to CF, was explored in KIRC in detail. Subsequent to that, an analysis was conducted to explore the variations of FUT8 expression within KIRC samples exhibiting diverse clinical attributes. Also, the molecular features of FUT8 were researched in a single-cell level. Consequently, we found that the state of CF was different in KIRC samples and was linked to the prognosis, immune response and drug sensitive of KIRC. All the findings in the research indicated that CF is a potential indicator of the TME in KIRC and regulating CF might be an approach to improve the prognosis of KIRC.

## MATERIALS AND METHODS

### Data acquisition and preprocessing

The genes involved in the process of the CF were collected and downloaded from the GeneCards database. For pan-cancer analysis, The Cancer Genome Atlas (TCGA) database was searched and the mRNA expression, survival data, single-nucleotide variation (SNV), and copy number variation (CNV) of common cancers were downloaded. In addition, the transcriptome profilings and clinical data of KIRC were obtained from both The Cancer Genome Atlas (TCGA) and the ArrayExpress database. Totally, the TCGA dataset contained 539 KIRC samples and the ArrayExpress dataset contained 101 KIRC samples. The batch effects between the two datasets were removed the utilizing the “sva” package in R [23, 24]. Prognostic core fucosylation-related genes (PCFGs) for KIRC were pinpointed through the implementation of univariate Cox regression analysis. Then these PCFGs were selected in the following in-depth analyses. Common immune checkpoint genes (ICGs) were summarized according to published research [25]. Genes participating in the signaling pathways were sourced from The Molecular Signatures Database (MSigDB).

### Pan-cancer analyses of CF

In view of the influence of the abnormal CF on the invasiveness, malignancy, and drug resistance of tumor cells in various cancers [26–29], the PCFGs which were CF-related hub genes were analyzed and summarized in pan-cancer. Initially, the fold change (FC) value was computed to evaluate the changes in gene expression between normal and tumor tissues in each cancer. Then, the univariate Cox regression analysis was performed to identify the prognostic value of each gene. Subsequently, the cumulative copy number variation (CNV), both amplified and deleted, was tallied. In the case of single nucleotide variation (SNV), the mutation frequency (number of samples with SNV divided by the total number of samples) was computed. The outcomes were visualized using a heatmap presentation. All these methods above have been summarized and utilized in previous studies [30–33].

### CF-based cluster analysis and identification of distinct KIRC subtypes

Subsequent sections involved comprehensive analyses aimed at delving into the potential significance of core fucosylation (CF) in the context of KIRC. The “ConsensusClusterPlus” packages in R was utilized to identify distinct KIRC subtypes. The various features between different KIRC subtypes were further explored. First, the “survival” package was utilized to plot the survival curves. Then the “ssgsea” function in “GSVA” package was chosen to estimate the state of CF. Broadly speaking, the “estimate” package was employed to evaluate aspects like tumor purity, immune activity, and stromal characteristics. As for estimating the TME specifically, the following approaches were utilized: (1) multiple algorithms including XCELL, MCPCOUNTER, CIBERSORT, CIBERSORT-ABS, EPIC, and TIMER were applied respectively to assess immune responses [34]; (2) the immune-related processes were estimated by “‘ssGSEA”; (3) the expression levels of common ICGs were collected and compared; (4) the condition of signaling pathways, encompassing those pertinent to immunity and metabolism, was gauged using the “ssgsea” method; (5) the states of common cell deaths were also estimated through “ssgsea”. Finally, the drug sensitivity in different KIRC subtypes was investigated by “oncoPredict”. Of note, the exploration of all the discrepancies between different KIRC subtypes was utilized “wilcox.test”.

### Subgroup analyses of FUT8 in KIRC

Fucosyltransferase 8 (FUT8), belonging to the fucosyltransferase family, assumes a pivotal role as the master controller in the core fucosylation process [35]. Till now, FUT8 has been outlined as an essential factor in the therapy of many cancers, such as liver, lung, colorectal, prostate, ovarian, breast [36]. But the potential influence of FUT8 in KIRC has not been well investigated. In the following research, we focused on the alteration FUT8 and its correlation with the tumor malignancy in KIRC. Utilizing the Biomarker Exploration for Solid Tumors (BEST) web server, which leverages extensive datasets, an investigation was conducted into the expression patterns of FUT8 across KIRC samples exhibiting diverse clinical attributes, with particular emphasis on tumor grade. With the increasing expression of FUT8, the altered gene mutation and CNV were also explored. In addition, the FUT8-related biological processes including GO and KEGG were summarized.

### Immunohistochemistry

Human tissue microarray sections of KIRC were procured from Zhuoli Biotech (Shanghai). Immunohistochemistry (IHC) was conducted by initially deparaffinizing and rehydrating the slides. Subsequently, tissue sections were incubated overnight with primary antibodies targeting FUT8. Following incubation with secondary antibodies, the sections were stained using diaminobenzidine and counterstained with instant hematoxylin. KIRC and normal tissues with complete IHC morphology were meticulously chosen for analysis. Two independent pathologists evaluated IHC scores based on staining intensity and the percentage of positive-stained cells.

### The assessment of the CF state based on scRNA-Seq data

KIRC scRNA-seq data (GSE156632) were retrieved from the GEO database and analyzed using the standard protocols of Seurat. Cells with less than 200 or more than 6500 count features were excluded, and cells with a mitochondrial RNA percentage exceeding 10% were also removed. Subsequently, the data underwent normalization and scaling for PCA analysis. The harmony package was employed to mitigate batch effects, and the “FindClusters” function was utilized to cluster cells at an optimal resolution. UMAP was employed for data visualization. Using typical cell-type markers, all subpopulations were identified and annotated. The CF state was assessed using five widely recognized algorithms: GSVA, UCell, singscore, Add, and AUCell. Importantly, the scores from the aforementioned five algorithms were aggregated to derive a total score, referred to as “Scoring.” Subsequently, the “wilcox.test” was employed to compare the CF state between KIRC and normal samples within each single-cell subpopulation.

### Availability of data and materials

The datasets analyzed in this work can be retrieved from public platforms. Also, any raw data and analytic technologies can directly contact the corresponding author and first author if the request is reasonable.

## RESULTS

### Data acquisition and identification of CF-related hub genes

All the KIRC samples were obtained from the TCGA and the ArrayExpress databases (TCGA: 539 samples; ArrayExpress: 101samples). After taking an intersection of the genes in the two datasets, the expression of 17612 genes were obtained. In total, a collection of 142 genes associated with core fucosylation were acquired from the GeneCards database. 62 CF-related hub genes with prognostic values were distinguished from the 142 genes after conducting univariate Cox regression analysis. In addition, 40 ICGs were collected for further analysis.

### Pan-cancer analysis based on CF-related hub genes

**Figure 1:**
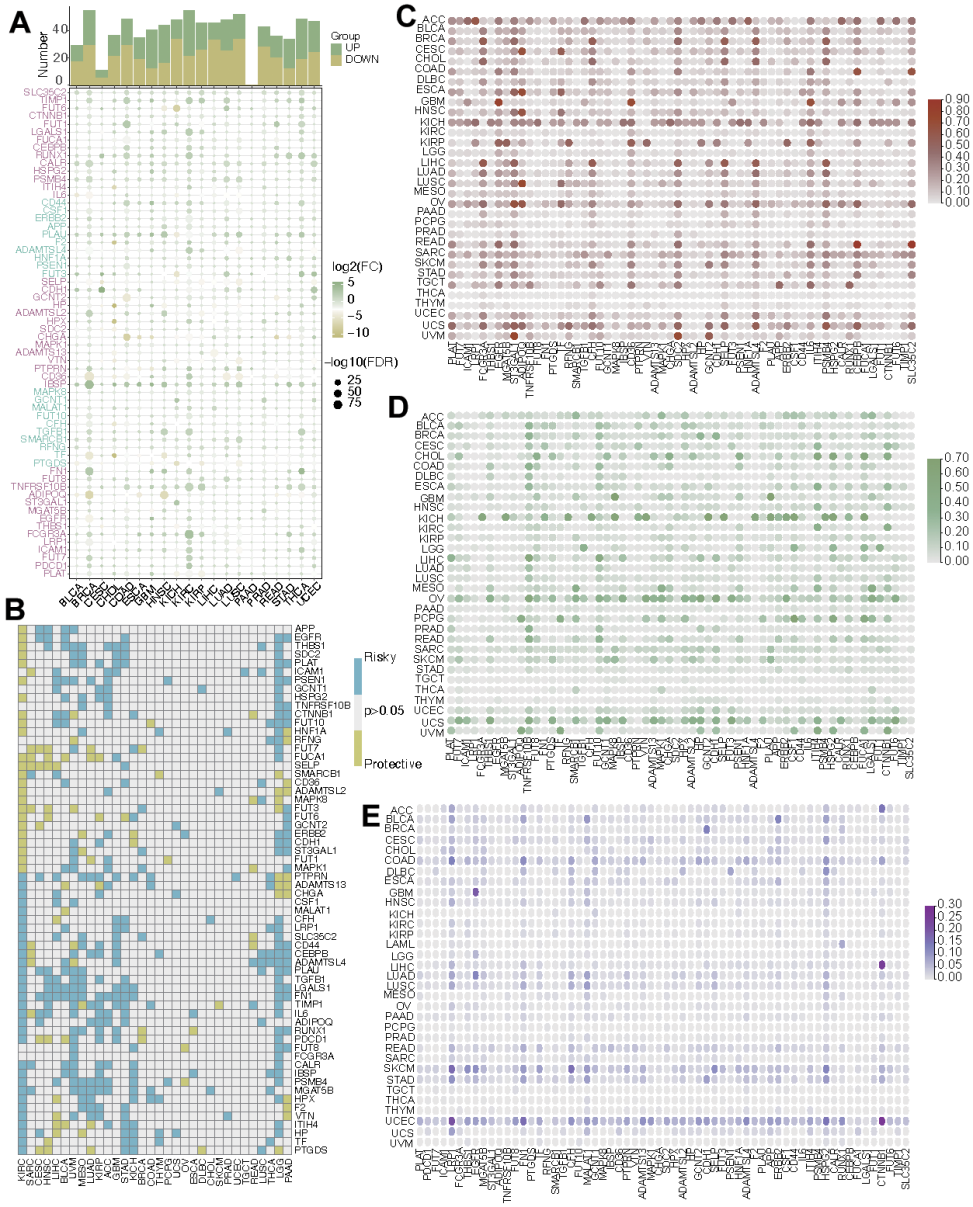
**Pan-cancer analysis of CF-related hub genes.** (**A**) The mRNA alteration, (**B**) the prognostic value, (**C**) the CNV gain, (**D**) the CNV loss, and (**E**) the SNV frequency of CF-related hub genes.

According to reports, core fucosylation (CF) occupies a crucial role in the initiation of tumorigenesis across various types of cancers. The alterations of the CF-related hub genes were summarized in pan-cancer. It was found that over half of the CF-related hub genes had increasing expression levels in tumor samples in many cancers including GBM, KIRC, STAD, and THCA (Figure 1A). Then the univariate Cox regression analysis was utilized to distinguish risky genes (HR>1, *p*<0.05) and protective genes (HR<1, *p*<0.05). The prognostic significance of every CF-related hub gene was evaluated and depicted through a heatmap visualization (Figure 1B). CNV, an important source of genetic variation, is a leading cause of the altered gene expression. The CNV gain mainly existed in ACC and KICH and the CNV loss primarily existed in OV and UCS (Figure 1C, 1D). SNV can also affect the gene expression levels. The SNV mutation frequency was higher in UCEC. Especially, LRP1, FN1, and CTNNB1 had obviously high SNV mutation frequency (Figure 1E).

### CF-based cluster analysis and identification of two KIRC subtypes

**Figure 2:**
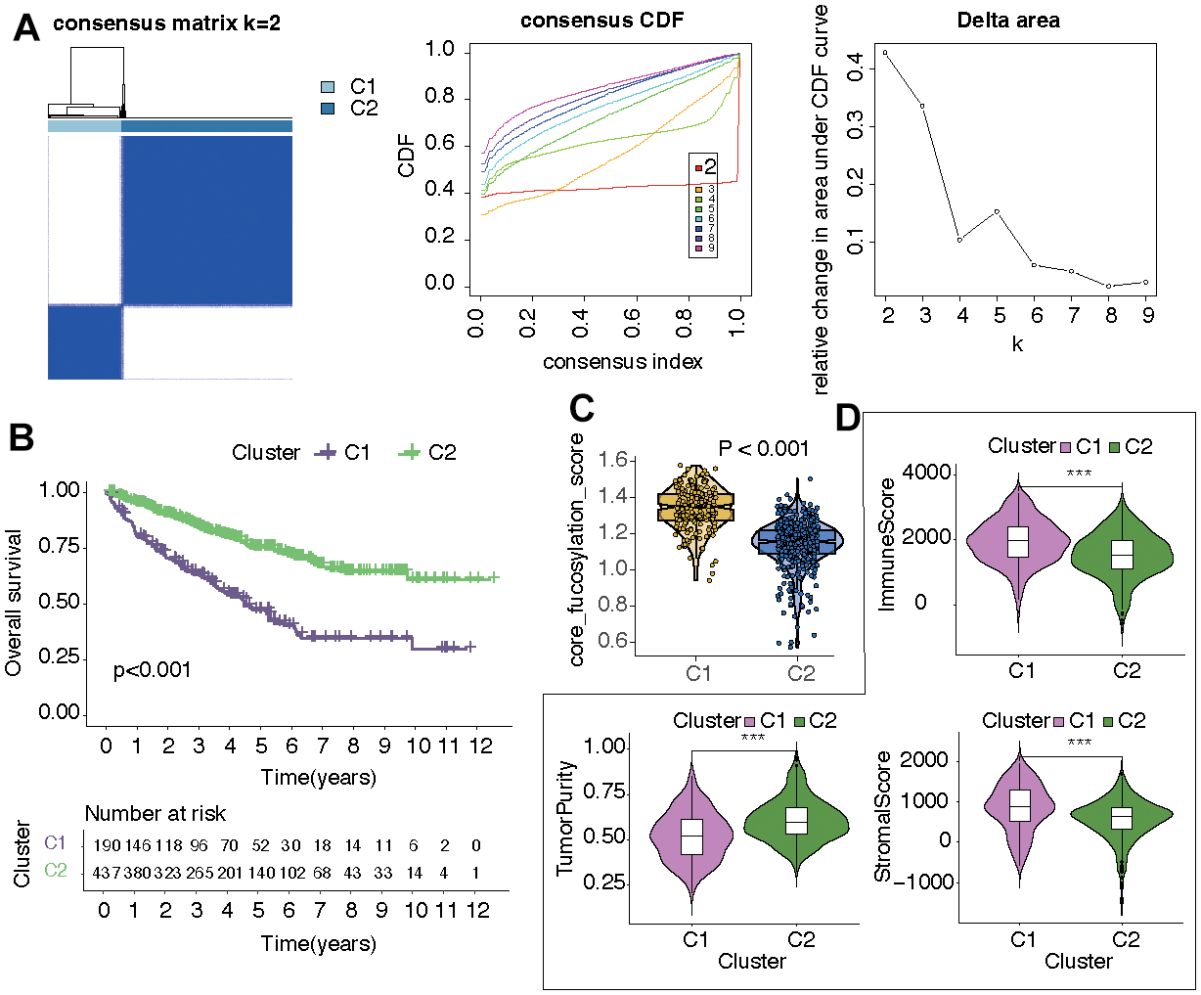
**Identification of two CF-related KIRC subtypes.** (**A**) Subtype distinguishment, (**B**) survival curves, (**C**) CF state, and (**D**) TME estimation of the two KIRC subtypes.

In the following part, the potential role of CF was explored in KIRC. Initially, all samples from KIRC were categorized into two distinct clusters, denoted as C1 and C2, relying on the expression levels of the CF-related hub genes (Figure 2A). The survival analysis suggested that samples in C1 had worse overall survival than that in C2 (Figure 2B). Next, the states of CF in the two clusters were estimated and compared. It was indicated that the CF score exhibited a higher value in cluster C1 compared to cluster C2 (*p*<0.001) (Figure 2C). As for the TME in the two clusters, in cluster C1, both the immune score and stromal score displayed elevated values in contrast to cluster C2, while the tumor purity in cluster C1 was comparatively lower than that observed in cluster C2 (Figure 2D).

### The variations in the immune microenvironment of the two KIRC subtypes

**Figure 3:**
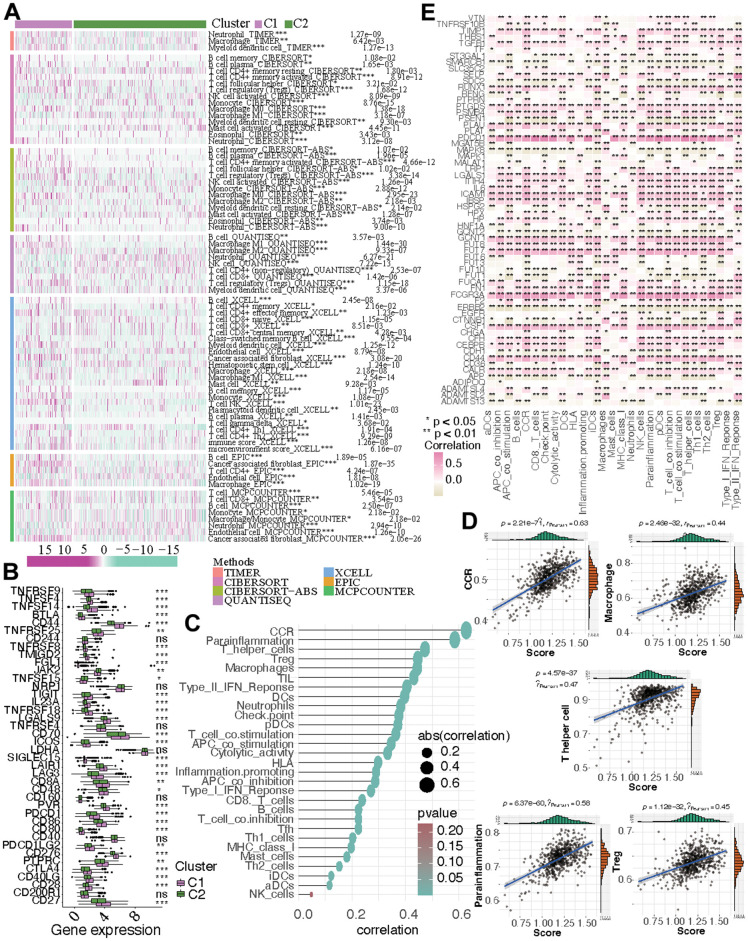
**The immune microenvironment in the two subtypes.** (**A**) The discrepancies of immune response in the two KIRC subtypes. (**B**) The altered expression of ICGs. (**C**, **D**) The correlation between immune-related process and CF score. (**E**) The correlation between immune-related process and the expression of CF-related genes.

First, the immune responses within the two subtypes were scrutinized, revealing dissimilarities in the levels of immune cell infiltration between them. Neutrophils, macrophages, myeloid dendritic cells, B cells, and T cell CD4+ memory activated were more active in C1, while mast cells, NK cells, and endothelial cells were more active in C2 (Figure 3A). As for the ICGs, the discrepancies between the two KIRC subtypes were shown in Figure 3B. Compared with C1, the expression of most ICGs (i.e., TNFRSF9, TNFSF4, TNFSF14, BTLA, CD44, TNFRSF25, TNFRSF8, TMIGD2, FGL1, TIGIT, IL23A, TNFRSF18, LGALS9, CD70, ICOS, SIGLEC15, LAIR1, LAG3, CD8A, CD48, PVR, PDCD1, CD86, CD80, PDCD1LG2, CD276, PTPRC, CTLA4, CD40LG, CD28, CD27) were decreasing while JAK2 and TNFSF15 had higher expression in C2. Certainly, the immune-related processes and immune cells were correlated with the state of CF. CCR and parainflammation were strongly correlated with CF score (r>0.5, *p*<0.05) and Cytolytic activity, APC co-stimulation, T cell co-stimulation, pDCs, checkpoints, Neutrophils, DCs, Type II IFN Response, TIL, Macrophages, Treg, and T helper cells were moderately correlated with CF score(r>0.3, *p*<0.05) (Figure 3C, 3D). Furthermore, the correlations between CF-related hub genes and the immune-related processes were explored. It was shown in Figure 3E that the expression levels of FCGR3A and PDCD1 had strong positive correlations with almost all immune processes. The expression of SELP was strongly correlated with Type II IFN Response. In addition, the expression of FUT7 displayed a substantial positive correlation with several factors, including TIL, pDCs, T cell co-stimulation, chemokine receptor (CCR), check points, T helper cells, Th1 cells, and inflammation-promoting (r>0.5, *p*<0.05).

### The discrepancies of the pathway activities and drug sensitivities between the two KIRC subtypes

**Figure 4:**
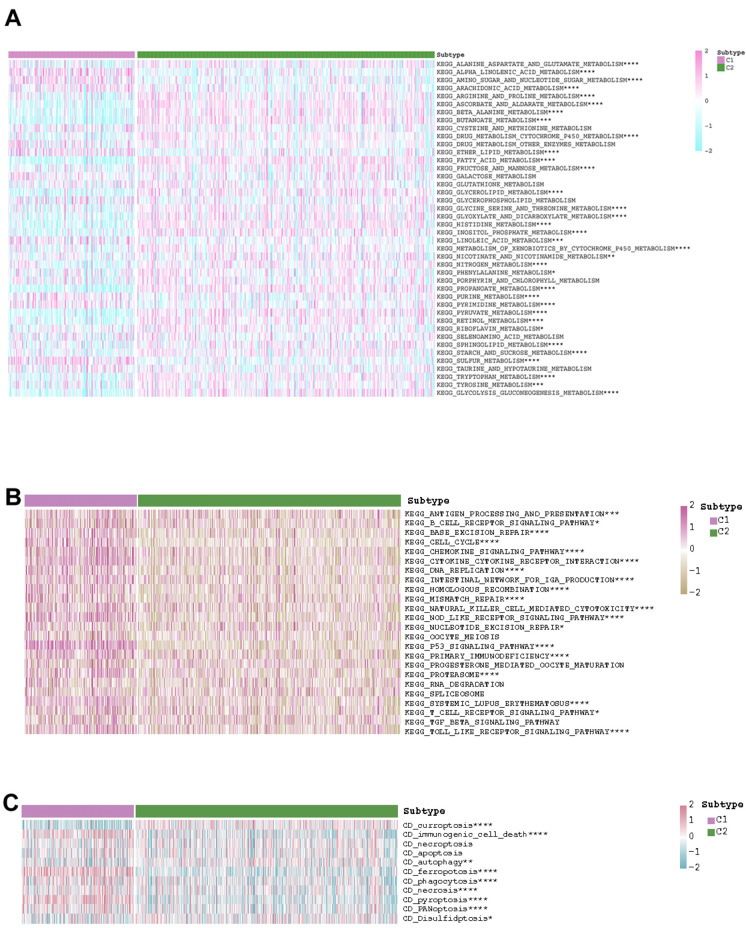
The exhibition of the distinct (**A**) metabolism-related pathways, (**B**) immune-related pathways, and (**C**) cell deaths in the two KIRC subtypes.

**Figure 5:**
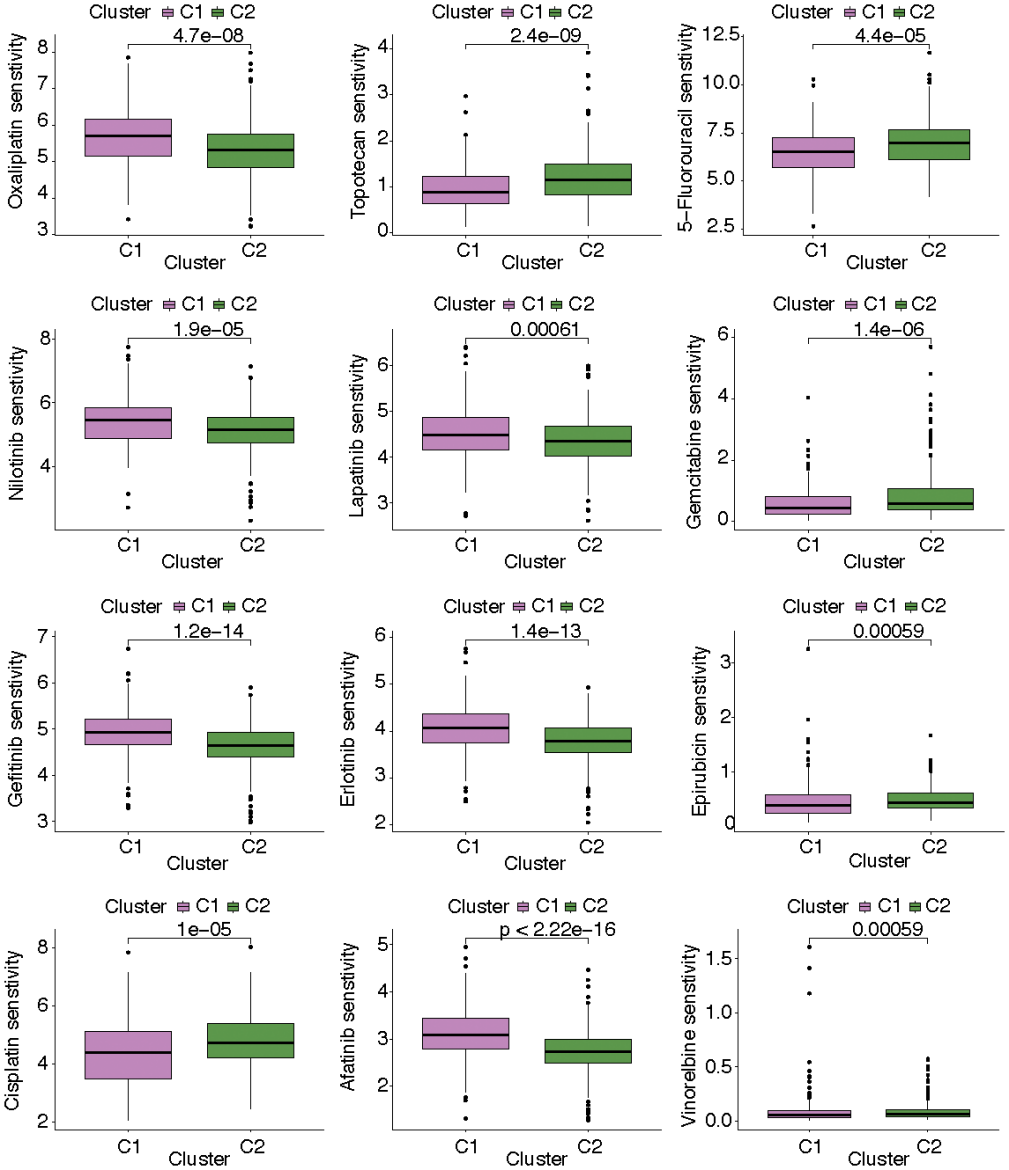
The discrepancies of drug sensitivity in the two KIRC subtypes.

After estimating the pathway activity, the discrepancies of the metabolism- and immune-related pathways and cell deaths were shown in the form of heatmap. In C1, the activities of alpha linolenic acid metabolism, amino sugar and nucleotide sugar metabolism, arachidonic acid metabolism, ether lipid metabolism, purine metabolism, pyrimidine metabolism, taurine and hypotaurine metabolism, and sulfur metabolism were up-regulated. In C2, the activities of arginine and proline metabolism, ascorbate and aldarate metabolism, beta alanine metabolism, butanoate metabolism, fatty acid metabolism, glycerolipid metabolism, glyoxylate and dicarboxylate metabolism, histidine metabolism, inositol phosphate metabolism, propanoate metabolism, pyruvate metabolism, retinol metabolism, glycolysis gluconeogenesis metabolism, and mTOR signaling pathway metabolism were up-regulated (Figure 4A). As for immune-related pathway, we found that the increasing activities of almost all immune-related pathways (i.e., antigen processing and presentation, B cell receptor signaling pathway, base excision repair, cell cycle, chemokine signaling pathway, cytokine cytokine receptor interaction, DNA replication, intestinal network for iga production, homologous recombination, mismatch repair, natural killer cell mediated cytotoxicity, NOD like receptor signaling pathway, nucleotide excision repair, oocyte meiosis, p53 signaling pathway, primary immunodeficiency, progesterone mediated oocyte maturation, proteasome, RNA degradation, spliceosome, systemic lupus erythematosus, T cell receptor signaling pathway, TGF beta signaling pathway, Toll like receptor signaling pathway) existed in C1 (Figure 4B). The state of cell death in the two subgroups also differed from each other. The state of curroptosis was more active in C2 while the state of immunogenic cell death, ferropotosis, phagocytosis, necrosis, pyroptosis, and PANoptosis were more active in C1 (Figure 4C). What’s more, the two KIRC subtypes showed distinct drug sensitivities. Samples in C2 may be more sensitive to vinorelbine, epirubicin, cisplatin, 5-fluorouracil, gemcitabine, and topotecan, while samples in C1 may be more sensitive to gefitinib, erlotinib, lapatinib, nilotinib, oxaliplatin, and afatinib (Figure 5).

### The investigation of the molecular features of FUT8 in KIRC through subgroup analysis and IHC

**Figure 6:**
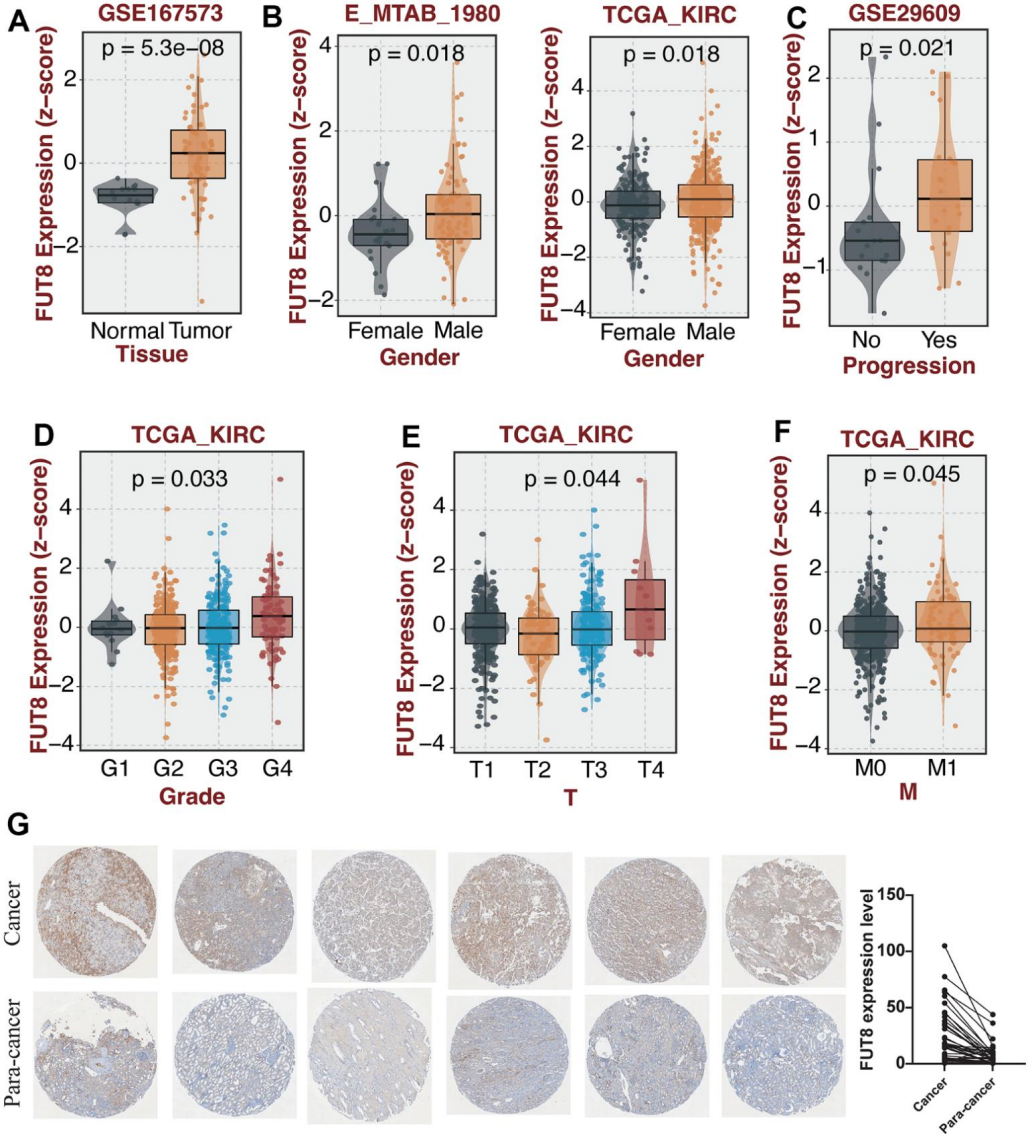
**The investigation of the molecular features of FUT8 in KIRC through subgroup analysis and IHC.** (**A**–**F**) The comparison of FUT8 expression in distinct KIRC subgroups. (**G**) Expression validation of FUT8 in KIRC and para-cancer.

FUT8, as the key regulator of CF, has the highest correlation score with CF in GeneCards. In view of the distinct features of CF in the two KIRC subtypes, the molecular features of FUT8 in KIRC were further investigated. Compared with normal samples, FUT8 had obviously higher expression in KIRC based on GSE167573 dataset (Figure 6A). Also, the expression level of FUT8 was found to be elevated in male KIRC patients compared to female patients, as evidenced by the data from both the E_MTAB_1980 and TCGA datasets (Figure 6B). In addition, its expression level was increasing in those KIRC samples with tumor progression based on GSE29609 dataset (Figure 6C). The expression of FUT8 showed highest level in G4 samples based on the TCGA dataset (*p*<0.05) (Figure 6D). As for T and M grade, we found that samples with T4 grade or M1 grade had the obviously high expression level of FUT8 based on TCGA dataset (Figure 6E, 6F). Subsequently, IHC revealed the expression level of FUT8 in KIRC and para-cancer tissues. Clearly, FUT8 exhibited higher IHC scores in KIRC compared to para-cancer tissues (p<0.05) (Figure 6G).

### FUT8-based enrichment analysis in KIRC

**Figure 7:**
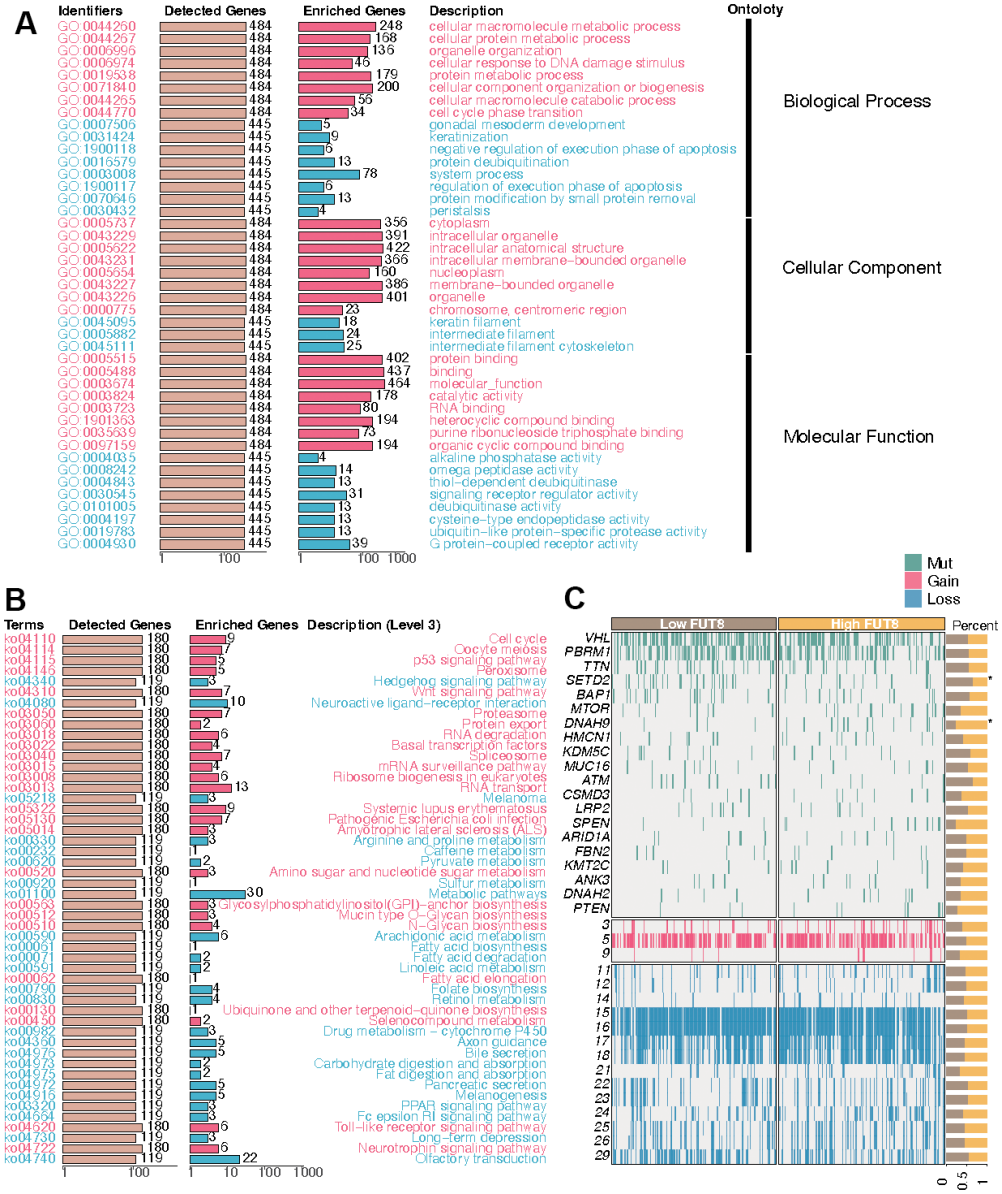
**FUT8-based enrichment analysis and mutation summarization in KIRC.** (**A**) GO enrichment, (**B**) KEGG enrichment, and (**C**) mutation in high-FUT8 and low-FUT8 subgroups.

Having designated FUT8 as the input variable, an over-representation analysis was conducted to identify the genes most closely linked to FUT8. Then the genes correlated with FUT8 were obtained and collected to carry out GSEA. First, the GO bar chart demonstrated that these genes exhibited enrichment across numerous biological processes (i.e., cellular macromolecule metabolic process, cellular protein metabolic process, organelle organization, cellular response to DNA damage stimulus, protein metabolic process), cellular component (i.e., cytoplasm, intracellular organelle, intracellular anatomical structure, intracellular membrane-bounded organelle, nucleoplasm), and molecular function (i.e., protein binding, molecular function, catalytic activity, RNA binding, heterocyclic compound binding) (Figure 7A). Then the KEGG bar chart suggested that these FUT8-related genes were correlated with many biological processes, such as cell cycle, p53 signaling pathway, hedgehog signaling pathway, wnt signaling pathway, mRNA surveillance pathway, arginine and proline metabolism, amino sugar and nucleotide sugar metabolism, arachidonic acid metabolism, drug metabolism-cytochrome P450, PPAR signaling pathway, and Toll-like receptor signaling pathway (Figure 7B). Additionally, with the increase of FUT8 expression, the genomics landscape of the TCGA-KIRC dataset was displayed in Figure 7C. We observed a higher frequency of SETD2 mutations within the low-FUT8 group and the mutation of DNAH9 was more frequent in high-FUT8 group.

### Identification of CF state based on single-cell transcriptional profiling

**Figure 8:**
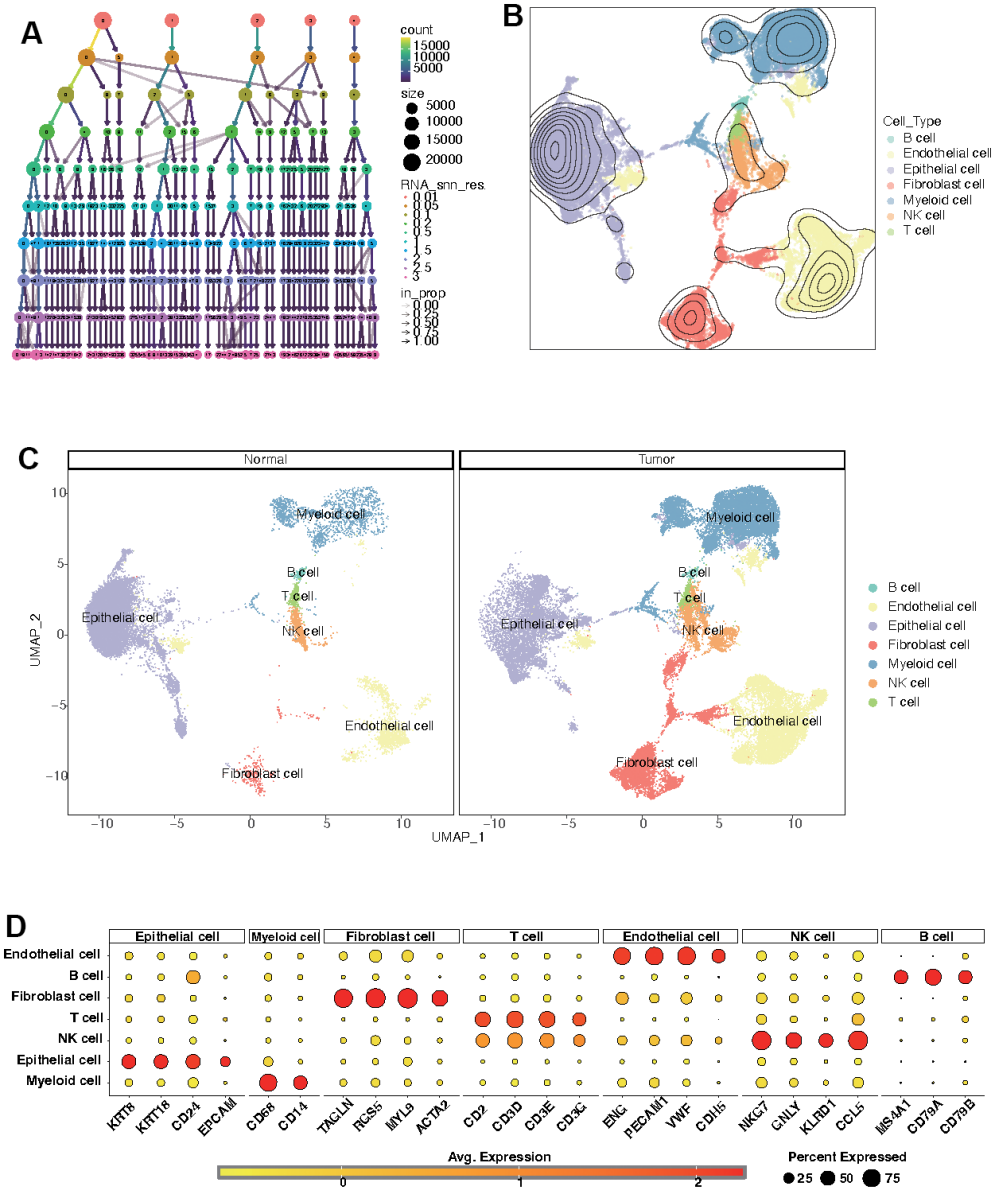
**Identification of distinct cell types in KIRC and normal samples.** (**A**) Clustree for identifying suitable cell clusters. (**B**) Various cell types across all samples. (**C**) Distinct cell types in KIRC and normal samples. (**D**) Expression of marker genes in each cell type.

**Figure 9:**
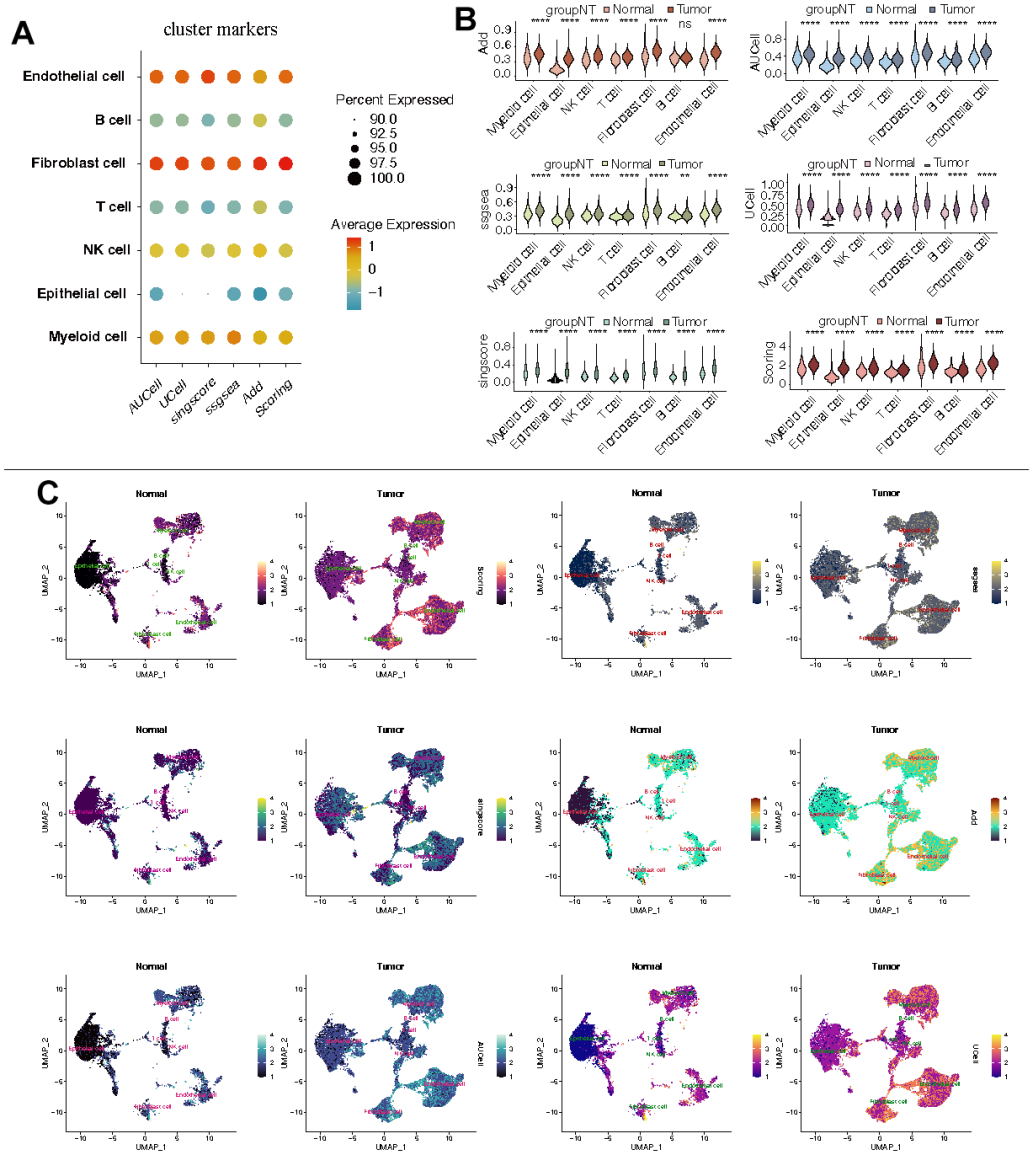
**Estimation of CF state based on scRNA-Seq data.** (**A**) CF state in each cell type; (**B**) Discrepancies of CF state in each cell type between KIRC and normal samples; (**C**) Detailed CF state depicted in a UMAP plot.

Further analysis of the scRNA-seq data (GSE156632) was performed to unveil the CF state in KIRC and normal samples. Following quality control (Supplementary Figure 1), all cells were grouped into 50 clusters (Figure 8A and Supplementary Figure 2A, 2B). Subsequently, these clusters were categorized into distinct cell types based on specific cell markers (Figure 8B–8D and supplementary Figure 2C). The CF state was evaluated in each cell type. It suggested that the CF state is notably active in endothelial and fibroblast cells (Figure 9A). The CF state of each cell type between KIRC and normal samples was compared, revealing statistical discrepancies in myeloid, epithelial, NK, T, fibroblast, and endothelial cells (Figure 9B, 9C).

## DISCUSSION

KIRC, combined with poor prognosis, is found to be easily resistant to chemotherapy and radiotherapy. Drug resistance can also occur in patients who received the target therapy. Absolutely, it is essential to identify the mechanisms of tumorigenesis and targets for tumor therapy in KIRC. Till now, the role of CF has been researched in many kidney diseases such as diabetic kidney disease and chronic kidney disease [37, 38]. Also, the latent influence of CF is explored from diagnosis to treatment in various cancers including liver, lung, colorectal, pancreas, and prostate cancers [26]. However, whether CF acts in the development and progression in KIRC is not well understood. In this research, we concentrate on the CF in KIRC and explored its potential role to the prognosis, TME, and drug therapy.

First, the PCFGs were singled out from the entirety of CF-related genes within KIRC. Subsequently, using the expression levels of these genes, KIRC samples were categorized into two distinct subtypes, denoted as C1 and C2, each exhibiting unique characteristics. The results of the in-depth analyses revealed that the C1 with poor prognosis had more active CF state. Also, the two KIRC subtypes with distinct state of CF had different TME. In recent years, TME was paid increasing attention because of its significant role in immune response, therapy response, and distant metastasis [39–42]. Research indicates that CF dysfunction can alter the TME. In lung cancer, FUT8 was identified as capable of regulating the cancer-promoting potential of cancer-associated fibroblasts through the modification of EGFR core fucosylation [43]. Furthermore, different states of CF in KIRC result in diverse TME conditions. We found that neutrophils, macrophages, B cells, and T cell CD4+ memory activated were more active while mast cells, NK cells, and Endothelial cells were more silent in C1. Of note, it was reported that the intra-tumoral neutrophils ranged from zero to 289 cells/mm (2) tumor tissue had a statistical correlation with increasing tumor size in KIRC and it was recognized as a standalone prognostic determinant in cases of localized renal cell carcinoma [44]. In addition, Bromwich and colleagues discovered a correlation between heightened levels of CD4+ T-cell infiltration within tumors and an unfavorable prognosis. [45, 46]. As for macrophages, it suggested that M1 macrophages had an association with a favorable prognosis while M2 macrophages linked with a poor prognosis in kidney cancer [47]. High density of CD20-defined B-cells indicated a poor-prognosis subset of RCC [48]. Mast cells might be diminished in KIRC which resulted in a weaken anti-tumor immune response [49]. The NK cells in KIRC were revealed to be correlated with the expression of TACC3 which was discerned as an autonomous risk element influencing prognosis [50]. The crucial role of endothelial cells in KIRC had been explored and an endothelial-related prognostic signature including three genes (i.e., CCND1, MALL, VWF) was constructed [51].

Additionally, there are also other discrepancies in the two KIRC subtypes, which may be related to the distinct state of CF. As is known, blocking the immune checkpoint pathways to hinder cancer cells from masquerading as constituents of the human body is a promising approach to realize anti-cancer immunity [52]. Our investigation revealed discernible differences in the expression levels of ICGs between the two subtypes of KIRC. Compared with C2, the expression of most ICGs (i.e., TNFRSF9, TNFSF4, TNFSF14, BTLA, CD44, TNFRSF25, TNFRSF8, TMIGD2, FGL1, TIGIT, IL23A, TNFRSF18, LGALS9, CD70, ICOS, SIGLEC15, LAIR1, LAG3, CD8A, CD48, PVR, PDCD1, CD86, CD80, PDCD1LG2, CD276, PTPRC, CTLA4, CD40LG, CD28, CD27) were increasing while JAK2 and TNFSF15 had lower expression in C1. These alterations might be novel targets to improve anti-cancer immunity. Additionally, the activities of metabolism- and immune-related pathways were distinct in the two KIRC subtypes. In C1, the activities of various metabolism-related pathways differed from those in C2, including pathways such as alpha linolenic acid metabolism, amino sugar and nucleotide sugar metabolism, and arachidonic acid metabolism. Moreover, there was a notable increase in the activities of nearly all immune-related pathways in C1, including pathways such as antigen processing and presentation and B cell receptor signaling pathway. The state of cell death also altered in the two clusters. Furthermore, the two KIRC subtypes showed distinct drug sensitivities. Samples in C2 may be more sensitive to vinorelbine, epirubicin, cisplatin, 5-fluorouracil, gemcitabine, and topotecan, while samples in C1 may be more sensitive to gefitinib, erlotinib, lapatinib, nilotinib, oxaliplatin, and afatinib.

FUT8 is demonstrated to be located on chromosome 14q24.3 and its special chromosome location which is distinct from other fucosyltransferase genes implies its unique biological significance [53]. The upregulation of FUT8 exists in many cancers [36, 54]. The expression level of FUT8 holds potential as a biomarker possessing either prognostic or diagnostic significance in many cancers including prostate cancer [55], pancreatic cancer [56], gastric cancer [57], and colorectal cancer [58]. Furthermore, our findings corroborated previous research by demonstrating an upregulation of FUT8 expression in KIRC. In the subgroup analysis, we found that increasing expression of FUT8 occurred in G4 samples, male patients, and those samples with T4 grade or M1 grade. All the results implied that FUT8 might act as a risk indicator in KIRC. Then, the IHC was conducted, revealing that FUT8 exhibited higher IHC scores in KIRC compared to para-cancer tissues. Besides, we found that the mutation of SETD2 was more frequent in low-FUT8 group and the mutation of DNAH9 was more frequent in high-FUT8 group. SETD2 was identified as one of the chromosome 3p21 epigenetic tumor suppressors and its mutation frequency in KIRC was 7.4% and 11.6% in the MSKCC and the TCGA cohorts respectively [59]. The exploration about DNAH9 mutation had little or no reports in KIRC. However, it has been recognized as among the ten most frequently mutated genes in cases of hepatocellular carcinoma [60] and the loss of the wild type allele of DNAH9 identifying a suppressor in esophageal squamous cell carcinoma [61]. What’s more, FUT8 inhibition can alleviate the cancer radioresistance and suppress the growth of tumor cell [62]. FUT8 is also reported to participate in humoral immune responses. FUT8 is associated with the maintenance of cancer cell stemness. It is found that the deficiency of FUT8 can down-regulate the stemness of cancer cells and result in the decrease of the expression of corresponding biomarkers, such as CD133, EpCAM, and c-Met [63–65]. All the findings above suggests that the regulation of FUT8 might a novel approach in the therapy in KIRC.

Finally, there are still a few drawbacks in our research which need to be optimized if possible. The current research was based on the analyses of retrospective data. Supplemental basic experiments are required for additional demonstration. Besides, ample clinical cohorts are essential to confirm our findings.

## CONCLUSIONS

In the research, two CF-related subtypes were identified based on the CF-related genes and the FUT8-related subgroup analyses were conducted in KIRC. The distinct state of CF may induce various TME, such as immune response and ICG expression. FUT8, as the key regulator of CF, was found to have an increasing expression in advanced KIRC samples, which implies it negative role in KIRC.

## Supplementary Material

Supplementary Figures
