# Supplementary material for: Identification of molecular characteristics of FUT8 and alteration of core fucosylation in kidney renal clear cell cancer
Source: Aging (Albany NY). 2024 Jan 25;16(3):2299–319. doi: 10.18632/aging.205482 (PMC10911337; doi:10.18632/aging.205482)
Supplement: Supplementary Figures [file aging-16-205482-s001.pdf]

SUPPLEMENTARY FIGURES

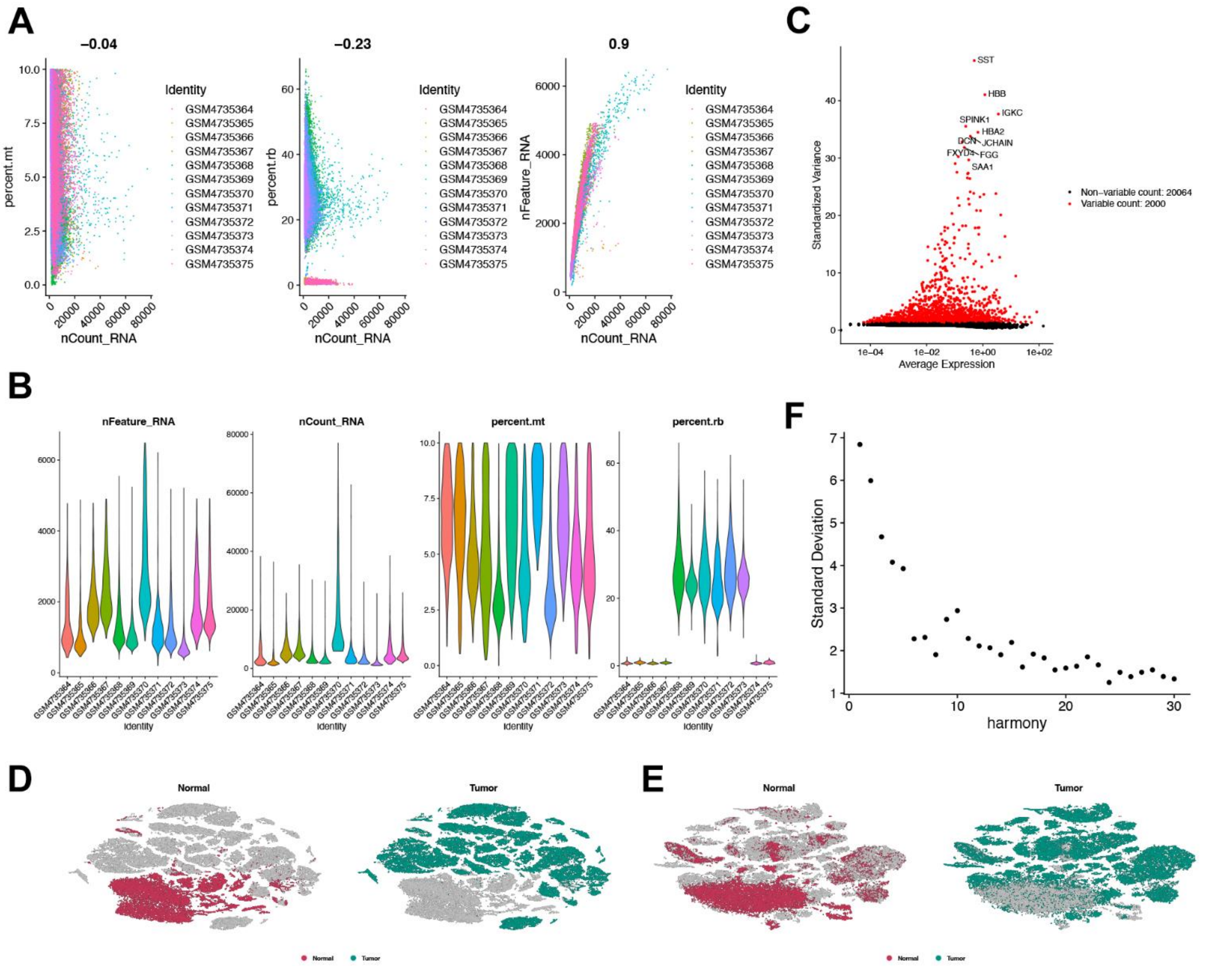

Supplementary Figure 1. Single-cell data processing process. (**A**, **B**) Quality control of the scRNA-seq data. (**C**) Highly variable genes in the data. (**D**, **E**) Cell distribution before and after removing the batch effect with Harmony. (**F**) ElbowPlot for identifying an appropriate number of principal components.

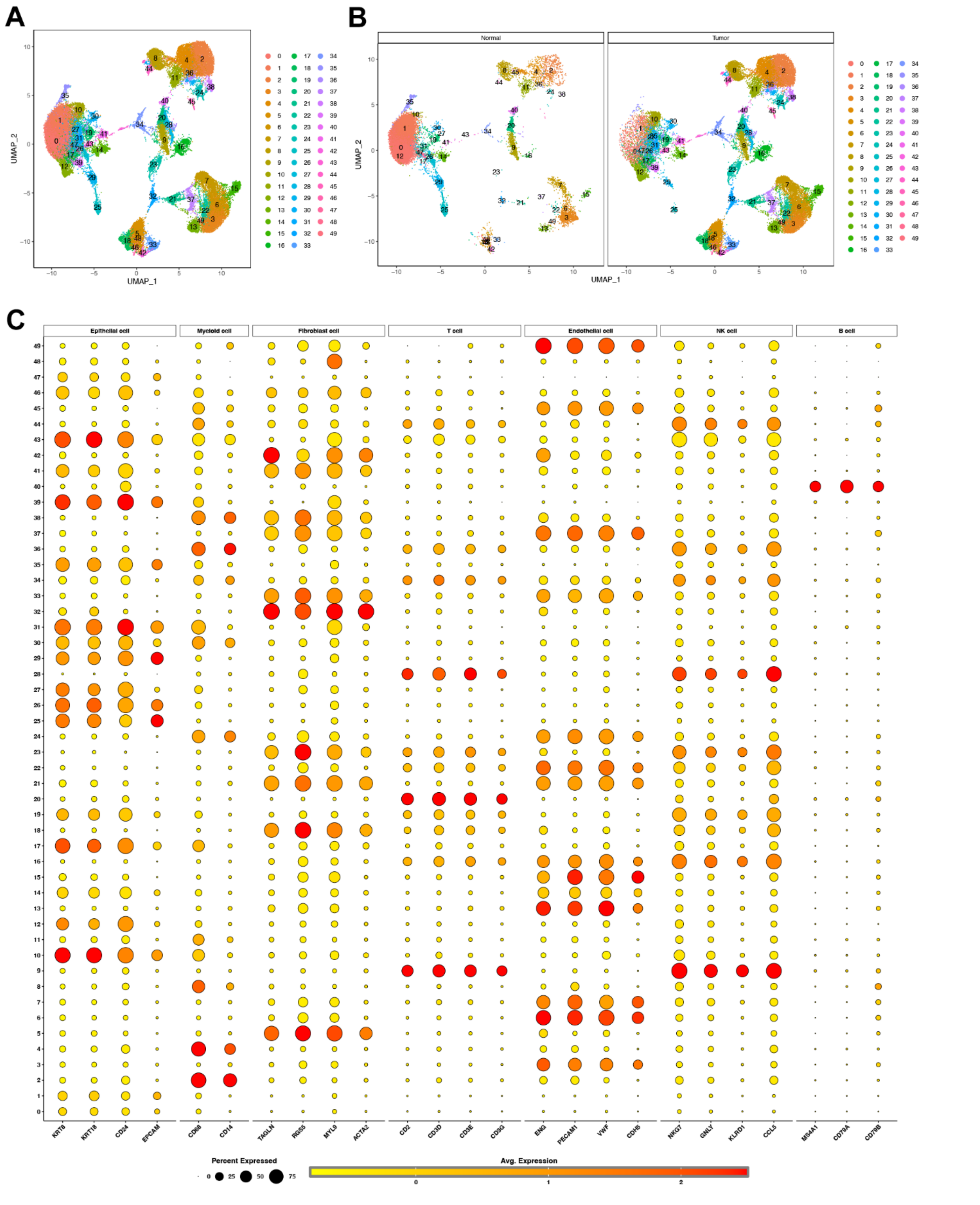

Supplementary Figure 2. Single-cell dimension reduction and cell annotation. (**A**) UMAP plots for all samples. (**B**) UMAP plots for normal and tumor groups. (**C**) The expression of cell markers in each cluster.
